# Questionnaire-based study of COVID-19 vaccination induced headache: evidence of clusters of adverse events

**DOI:** 10.1186/s12883-024-03583-6

**Published:** 2024-03-02

**Authors:** Qiao Zhou, Thomas Eggert, Ana Zhelyazkova, Alexander Choukér, Kristina Adorjan, Andreas Straube

**Affiliations:** 1 Department of Neurology, University Hospital, LMU Munich, Marchioninistr.15, 81377 Munich, Germany; 2 Institut für Notfallmedizin und Medizinmanagement, Klinikum der Universität München, 80336 Munich, Germany; 3 Laboratory of Translational Research Stress and Immunity, Department of Anaesthesiology, University Hospital Munich, Ludwig Maximilian University of Munich, 81377 Munich, Germany; 4 Department of Psychiatry and Psychotherapy, University Hospital, LMU Munich, 80336 Munich, Germany

**Keywords:** Headache, Covid-19 vaccination, Adverse events, Side effects

## Abstract

**Background:**

The adverse events (AEs) after a Coronavirus disease 2019 (Covid-19) Pfizer-Biotech mRNA vaccination present a medical and epidemiological issue of increasing interest. Headache is the most frequent neurological adverse effect and generally the third most common adverse event after a Covid-19 vaccination, but only a few studies focus on the link between headache and other AEs after vaccination. This study aims to investigate the correlation between headaches and Covid-19 vaccination, as well as the possible links between headaches and other AEs after Covid-19 vaccination, thereby helping the management of AEs and avoiding further occurrences.

**Methods:**

This study is based on a published questionnaire survey of 1,402 healthcare workers. Our study focused on the 5 questions including 12 AEs and headaches extracted from the questionnaire post the first and second Covid-19 vaccination. The severity of the 12 AEs and headaches could be classified by the participants on a five-step scale: “Not at all”, “Little”, “Average”, “Quite”, and “Very” (abbreviated as “N”, “L”, “A”, “Q”, “V”). We used the Bowker test to study the comparison of headache severity, indicated on a 5-point Likert scale between the first and second vaccinations. We applied an ordinal logistic regression to the 5 categories with headache severity serving as the dependent variable and the ratings of the other 12 AEs serving as the independent variable to further explore to what extent the severity of the 12 AEs is associated with the severity of headaches. Receiver Operating Characteristic (ROC) analysis was conducted to evaluate the predictive value of the ratings of the 12 AEs to headache severity.

**Results:**

We found that participants rated their headaches as more severe after the second vaccination, and participants who reported experiencing fatigue, flu-like symptoms, pain at the injection site, known tension-type headache, fever, dizziness/balance problems and known migraine are associated with headache symptoms.

**Conclusions:**

There are clusters of headache-associated AEs post Covid-19 vaccination. The association of various AEs with headaches may be due to similar causative mechanisms.

**Supplementary Information:**

The online version contains supplementary material available at 10.1186/s12883-024-03583-6.

## Introduction

The Coronavirus disease swept rapidly throughout the world after its outbreak at the end of 2019 (Covid-19) [1]. To combat this pandemic, several targeted vaccines have been used globally with significant benefits in terms of reduced mortality and hospitalization rates [2]. In the majority of cases, the severity of SARS-CoV-2 infection exceeds the risk of self-limited adverse events (AEs), but it is still necessary to study the potential safety concerns. The AEs after the Covid-19 vaccination have recently received more attention. Common AEs that have been reported are fatigue, headache, myalgia, injection site pain, muscle pain and chills [3, 4].

As reported, AEs post Covid-19 vaccination can be classified as neurological symptoms (e.g. headache, anosmia, ageusia) [5], gastrointestinal tract-related symptoms [6], and cardiovascular and hematological-related symptoms [7]. Studies concerning influenza and herpes vaccination showed that headaches can be triggered by several types of vaccines and are the fifth most frequently reported AE overall [8]. Moreover, various vaccine-induced neurological sequelae have been elaborated recently, which include Covid-19 vaccine-induced headache [9]. A meta-analysis reported that headache was the most frequent neurological symptom and was generally the third most common AE after the Covid-19 vaccination. It further showed that 22% and 29% of participants had headaches after the first and second vaccination respectively within 7 days [10]. Moreover, migraine-like symptoms after receiving the Covid-19 vaccination have a wide range of accompanying symptoms, the most frequent of which are fatigue (38.8%), exhaustion (25.7%) and muscle pain (23.4%) [11]. Another study reported that post-vaccination AEs are associated with participant anxiety; the correlation between headache and higher anxiety are statistically significant [12].

Considering that headache is a potential diagnostic marker for mild AEs that occur immediately after vaccination or a warning sign of serious AEs post-vaccination (e.g., cerebral venous and sinus thrombosis [13]), we explore the difference and correlation between the headaches after the first and second Covid-19 vaccination and whether such a type of headache occurs in conjunction with other specific AEs.

## Materials and methods

### Data from the in-hospital vaccination center

This study is based on our previous questionnaire-based project, which has been published and focused primarily on the organization, program monitoring, and satisfaction of the participants in an in-hospital vaccination center [14], and the main intention of the project was to obtain an insight into the vaccination center in order to optimize it for future pandemics. During the vaccination campaign at the University Hospital of Ludwig Maximilian University in Munich Germany, all healthcare workers (HCWs) at the hospital were invited to participate in the free vaccination program. In addition, the HCWs were also asked to participate in an anonymous internet-based survey. 1,662 out of more than 11,000 HCWs were willing to participate in the survey on vaccination, and the questionnaire was filled out anonymously online, the vaccination type was a mRNA vaccine (Comirnaty©; Biotech/Pfizer) in all cases, more detailed information on vaccines and participants has been elaborated in the project before [14]. This study focused on the AEs post Covid-19 vaccination, especially the correlation between headaches and other AEs. The severity of the AEs could be classified by the participants on a five-step scale: “Not at all”, “Little”, “Average”, “Quite”, and “Very”. These five categories are abbreviated in the following as “N”, “L”, “A”, “Q”, “V”.

The questionnaire about the organization and procedure has been discussed previously [14]. The following questions about headache and AEs in the questionnaire (See supplementary file.) were extracted and included in our research:

Question1 (Q1): Did you observe any AEs after the first vaccination? (Yes/No)

Question2 (Q2): Did you observe any AEs after the second vaccination? (Yes/No)

Question (Q3): How do you rate the severity of headache after the first vaccination?

Question4 (Q4): How do you rate the severity of headache after the second vaccination?

Question5 (Q5): How do you rate the severity of the following 12 symptoms after the second vaccination?

Q5 addressed the following 12 AEs: (1) Fatigue, (2) Flu-like symptoms (aching limbs, chills), (3) Pain at the injection site, (4) Known tension headache (triggering an attack within 24 h), (5) Fever (≥ 38℃), (6) Dizziness/Balance problems, (7) Known migraine (triggering an attack within 24 h), (8) Circulatory weakness (dizzy/blackout), (9) Nausea/Vomiting, (10) Diarrhea, (11) Redness of injection site, (12) Hematoma.

### Statistical analysis

The sample size was based on the number of participants that submitted the questionnaire. Firstly, only the participants who answered both the first two questions Q1&Q2 in the questionnaire were included in further analysis. We analyzed whether there was a difference in the frequency of experienced AEs between the first and second vaccination using the McNemar test. We also used the Bowker test to study the comparison of headache severity, indicated on a 5-point Likert scale between the first and second vaccinations, and abstracted the data from the questionnaire Q3&Q4. Participants who responded to questions Q1&Q2 with “No” were counted as if they had answered questions Q3&Q4 with “Not at all”. Spearman rank correlation was used to evaluate the association of the headache severity between first and second vaccination. The binomial test was taken to compare the frequency of headaches post second vaccination among people who had headaches after first vaccination with people who did not. We further explore to what extent the severity of other 12 AEs (also indicated on the scale with 5 gradations) is associated with the severity of headache. Since the number of positive evaluations after the second vaccination was significantly higher than that after the first vaccination, the severity of AEs was evaluated only after the second vaccination. We applied an ordinal logistic regression on the 5 categories with headache severity serving as the dependent variable and the ratings of the other 12 AEs serving as independent variables. MATLAB (The MathWorks, Inc., Version 9.9.0 (R2020b) ) was taken to conduct the data analysis, function “mnrfit” for ordinal logistic regression which performs the same analysis as the function “polr” from the package MASS in R [15]. Receiver Operating Characteristic (ROC) analysis was conducted to evaluate the predictive value of the ratings of other AEs assessed by Q5 to headache severity. Based on the slopes of the ordinal regression, we also computed the predicted probability of each participant rating headache stronger than average (P (Q5 > “A”)) and generated a classifier to distinguish participants with a low and high headache rating. All methods carried out in the study were performed in accordance with relevant guidelines and regulations.

## Results

### Study population

A total of 1,662 participants filled out the questionnaire. Since the purpose of our study is to investigate the AEs of Covid-19 vaccines post-vaccination, we only included 1,402 participants who gave clear answers (Yes or No) to Q1&Q2 regarding whether they experienced AEs after first/second vaccination. The largest age group among the 1,402 participants is that of 30–59 years old, 22.7% of participants are male and 71.4% are female, the remaining study participants did not provide any information on gender.

### Adverse events after first and second vaccination

The McNemar test showed that the number of participants changing their response to question Q1 (including all the 12 AEs) from “No” after the first vaccination to “Yes” after the second vaccination (356) was significantly (*p* < 0.0001) larger than the number of participants with response changes in the opposite direction. Thus, the frequency of AEs was larger after the second dose (986/1402 = 70.3%) than after the first dose (728/1402 = 51.9%). (See Table 1.)

**Table 1:** Comparison of AEs frequency after vaccination between the first and the second dose

		Q2		Sum
		No	Yes	
Q1	No	318	356	674
	Yes	98	630	728
	Sum	416	986	1402

AEs were more frequent after the second vaccination. Question1: Q1, Question2: Q2. Sum: Summary

### Headache severity after first and second vaccination

Based on the first result on the difference in the frequency of AEs, we aimed to investigate the difference in headache severity between the first and the second vaccination. Table 2 shows that the number of participants who increased their headache rating after the second vaccination compared to that after the first vaccination (sum of the frequencies above the diagonal: 443) was much larger than the number of participants with rating changes in the opposite direction (sum of the frequencies below the diagonal: 115). This difference was statistically confirmed by the Bowker test (*p* < 0.0001).

Thus, participants rated their headaches as more severe after the second vaccination. To quantify this rating change, we coded the five rating levels to interval scaled numbers between 0 and 4 and computed the average across all participants. It was 0.41 after the first vaccination and 0.99 after the second vaccination.

Additionally, this result may also reflect the increased number of participants with headache after the second vaccination (Q4 > “N”). We further tested the additional hypothesis that all participants who had headaches after both vaccinations rated their headaches stronger after the second one (Q4 > Q3). This hypothesis was confirmed by repeating the Bowker test (*p* = 0.016) for the subset of cells in Table 2 with Q3 > “N” and Q4 > “N”.

**Table 2:** Comparison of the headache rating after the vaccination between the first and the second vaccination

			Q4: Rating after 2nd dose					Sum
			N	L	A	Q	V	
Q3	Rating after 1st dose	N	775	92	95	89	95	1146
		L	32	16	14	12	7	81
		A	20	9	22	16	10	77
		Q	8	4	12	12	13	49
		V	19	2	6	3	19	49
	Sum		854	12/3	149	132	144	1402

Participants experienced more severe headache after the second dose. Not at all, Little, Average, Quite, Very (abbreviated as “N”, “L”, “A”, “Q”, “V”). First: 1st. Second: 2nd

A total of 256 participants had headaches after the first vaccination (Q3 >“N”). Of those, significantly (Binomial test: *p* < 0.0001) more also had headache after the second vaccination (177 = 69.1%) compared to those who did not (79 = 30.9%), Spearman rank correlation indicates the significant association between headaches after first and second vaccination (rho = 0.3445, *P* = 0.000003), which means participants who had headaches post first vaccination have a higher chance of also having headaches after the second vaccination.

### Observation of the cluster adverse events

The frequencies of the ratings of the independent variables after the second vaccination are shown in Table 3. The first four AEs showed the largest frequencies of non-zero ratings (Rating > “N”).

**Table 3:** Absolute frequencies of AEs rating

AEs	N	L	A	Q	V	Sum
Pain at the injection site	603	222	268	183	126	1402
Fatigue	646	87	183	228	258	1402
Flu-like symptoms	851	106	107	137	201	1402
Headache	854	123	149	132	144	1402
Fever	1148	59	58	63	74	1402
Dizziness/balance problems	1181	75	58	52	36	1402
Redness	1188	122	56	17	19	1402
Circulatory weakness (dizzy/blackout)	1211	79	53	39	201	1402
Nausea/Vomiting	1274	49	36	25	181	1402
Known tension-type headache (triggering an attack within 24 h)	1298	205	15	37	32	1402
Diarrhea	1323	29	26	14	10	1402
Hematoma	1326	40	17	10	9	1402
Known migraine (triggering an attack within 24 h)	1347	12	7	14	22	1402

Not at all, Little, Average, Quite, Very: “N”, “L”, “A”, “Q”, “V”. Advent Events: AEs. Sum: Summary

The ordinal regression showed significant associations of the general headache rating (Q4) with the ratings of some but not all AEs. The ratings of fatigue, flu-like symptoms, pain at the injection site, known tension-type headache, fever, dizziness/balance problems, and known migraine attack within 24 h reached significance (see Table 4). This finding means that the occurrence of these 7 AEs but not of the others were statistically coupled with the occurrence of headache.

**Table 4:** Slopes fitted in the ordinal regression, their t-values, and false positive probabilities

Independent Variable	Fitted Slope	T-Value		alpha
Fatigue	${S}_{1}$= -0.52	T(5592)= -9.9		**< 0.001**
Flu-like symptoms	${S}_{2}$= -0.362	T(5592)= -7.1		**< 0.001**
Pain at the injection site	${S}_{3}$= -0.342	T(5592)= -6.5		**< 0.001**
Known tension-type headache (triggering an attack within 24 h)	${S}_{4}$= -0.462	T(5592)= -5.8		**< 0.01**
Fever	${S}_{5}$= -0.19	T(5592)= -3.2		**< 0.01**
Dizziness/balance problems	${S}_{6}$= -0.22	T(5592)=-2.8		**0.01**
Known migraine (triggering an attack within 24 h)	${S}_{7}$= -0.26	T(5592)=-2.6		**0.01**
Circulatory weakness(dizzy/blackout)	${S}_{8}$= -0.11	T(5592)=-1.2		0.23
Nausea, vomiting	${S}_{9}$= -0.09	T(5592)=-1.0		0.31
Diarrhoea	${S}_{10}$= 0.04	T(5592) = 0.4		0.68
Redness	${S}_{11}$= 0.02	T(5592) = 0.2		0.83
Hematoma	${S}_{12}$= >-0.01	T(5592)<-0.02		> 0.98

The first 7 AEs reached significance, which means there was an association between the rating of headache with these 7 AEs but not with the others

**Fig. 1:**
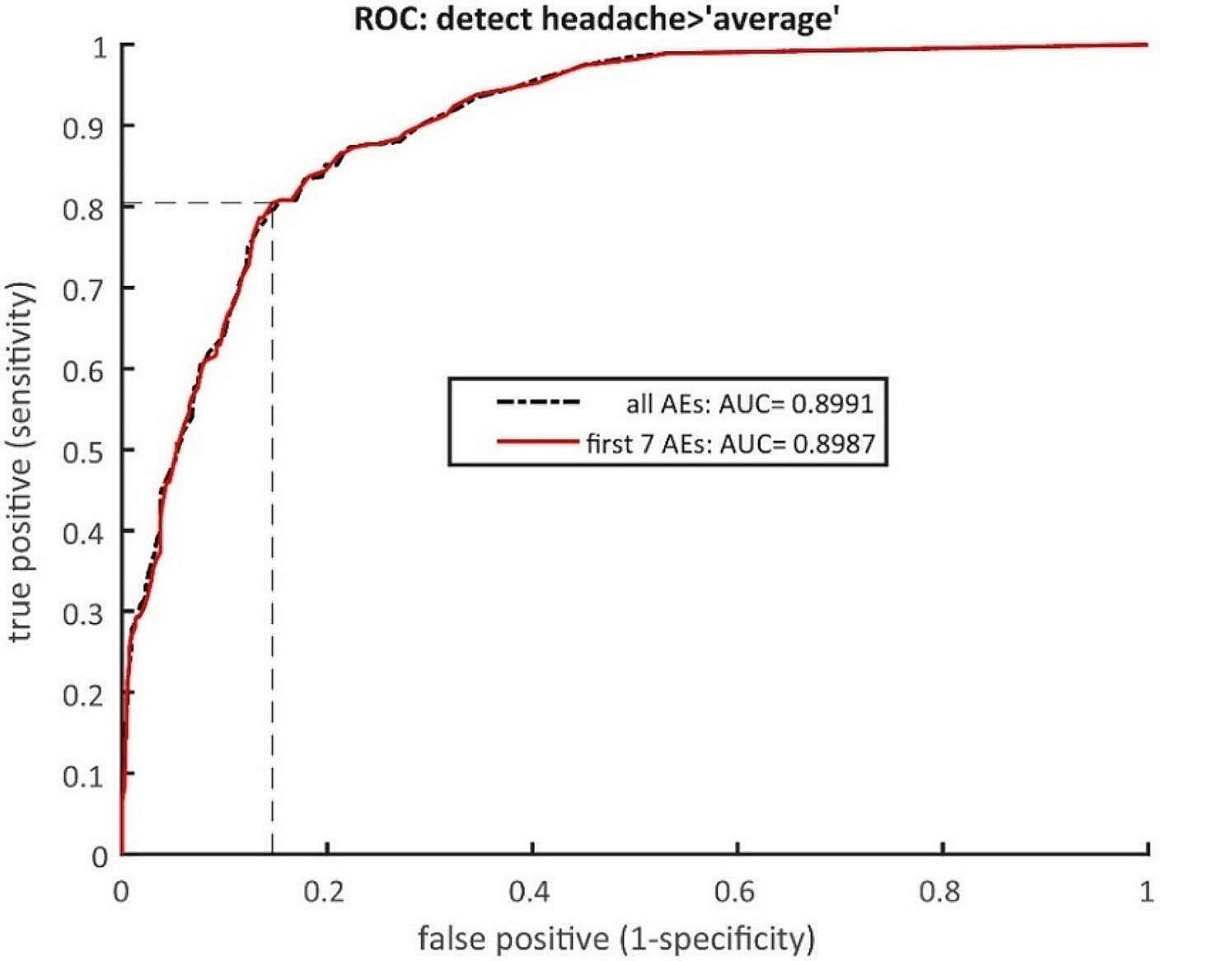
The ROC curve of the classification between high and low rates of headache. Using the first 7 AEs in Table 4 whose rating are related to headache severity, classification is optimal at a threshold 0. 25, with a false positive rate of 0.147 (vertical dotted line) and a true positive rate of 0.80 (horizontal dashed line). The fraction of correct responses was 0.8 and AUC was 0.90

The ROC analysis (See Fig. 1) used the ratings of the AEs (Q5) to predict the probability of each participant rating headache stronger than average (*P* (Q4 > “A”) on the basis of two ordinal regressions. The first included only the ratings of the first 7 AEs in Table 4 whose slope differed significantly from zero, and the second that of all AEs. Using the first 7 AEs only, classification was optimal at a threshold of 0.25, with a false positive rate of 0.147 (vertical dashed line in Fig. 1) and a true positive rate of 0.80 (horizontal dashed line in Fig. 1). The fraction of correct responses was 0.8 and the area under curve (AUC) was 0.90. Including the irrelevant AEs: circulatory weakness (dizzy/blackout), diarrhea, nausea, local redness and hematoma achieved the same fraction of correct responses (0.83) and thus did not improve the classification performance. The ratings of the AEs other than headache allowed a successful classification of the participants into high-raters and low-raters of headache. This demonstrates a strong dependence of the headache rating on the rating of a cluster of other AEs.

## Discussion

In the present study, we investigated the incidence of headaches and other AEs after the Covid-19 vaccination (Comirnaty©) in a population of HCWs (*N* = 1,402). Our results showed that participants had a significantly higher frequency of AEs in general, as well as an increased frequency of headache after the second vaccination compared with the first vaccination. Generally, the rating of the headache after the second vaccination was more severe than that after the first vaccination. In addition, subjects who developed headaches after the first vaccination tended to have more chance of getting headaches again after the second vaccination. Concerning the relation of headache after the first and the second vaccination, similar results were reported in a previous cross-sectional study [16], which showed an incidence of overall headaches of 20.5% and 45.6% after first and second vaccination, respectively. In the non-migrainous headache group of this study, the Numerical Pain Rating Scale score of the second dose-induced post-vaccination headache was significantly higher than that of the headache induced by the first dose. In our study, we extended this finding using the ROC analysis, which indicated that if participants have AEs such as fatigue, flu-like symptoms, pain at the injection site, known tension-type headache, fever, dizziness/balance problems and known migraine, they tend to rate their headache as more severe. This might be due to a specific effect, since other AEs such as circulatory weakness (dizzy/blackout), diarrhea, nausea, local redness and hematoma did not show such an association with headache ratings. This supports the idea that some but not other AEs show a specific relation to headache.

Recent data published by an Italian headache center based on another questionnaire revealed that 66.47% and 60.15% of patients with prior migraine experienced headache attacks after the first and second vaccination respectively, and worsening of prior headaches after the Covid-19 vaccination were seen [17]. Furthermore, patients with migraine reported a significantly higher headache pain intensity on the verbal rating scale after Covid-19 vaccination compared to participants who had no history of migraine [16, 18, 19], which poses the question whether there is a shared pathophysiology in some part of these headaches. This may point towards a greater susceptibility of patients with migraine history to respond to an inflammatory stimulus (e.g. vaccination) with headache. One questionnaire-based study revealed that the clinical characteristics after vaccination of HCWs like primary headache history, influenza vaccine-related headache, thyroid disorder, Covid-19-related headache, and female gender can be a predictor of vaccine-related headache [19], which may underline the relation of a headache history and the response with headache to inflammatory stimuli (e.g., influenza vaccination, inflammatory thyroid disorders).

### Covid-19 vaccine-related headache mechanisms

The pathophysiological mechanisms of vaccine-related headache are unclear. We observed that participants who have prior tension-type or migraine headache attacks can be triggered within 24 h after vaccination. Normally, the delay between the first contact with a pathogenic agent and the occurrence of antibodies (IgM type) is 5–8 days [20]. The short latency of headache after vaccination of 12–24 h favors an activation of the innate immune-system with the activation of NOD-like receptor protein 3 (NLRP3), which initiates a series of inflammatory events, compared with the adaptive immune system which needs much more time (5 to 7 days) in order to respond [21, 22]. Besides, headaches are more frequent after the second vaccination of mRNA vaccines, CD4 + T-cell expresses with Th1 cytokines like TNF-α, which can sensitize meningeal nociceptors and stimulate the synthesis of the migraine-associated calcitonin gene-related peptide [23].

The common neurological Covid-19 vaccine AEs documented before are headache (18.2%), fatigue (16.5%), muscle pain (16%), numbness (3%), and migraine (2.6%) [24]. Additionally, several studies also describe that headache is the second most commonly occurring systemic side event, following fatigue [25–27]. Beside headache, the most common AE is a local reaction at the injection side [28]. Our study gave a possible explanation for that concurrent frequency by demonstrating that when participants have severe fatigue and injection site pain, they tend to concurrently have severe headaches as well. The possible mechanism might be that the severity of fatigue in acute infection disease is influenced by genetic polymorphisms in NLRP3 and IL-1β [29]. Further, the central sensitization, which is an important mechanism for the emergence of chronic migraine, can also be influenced by the activation of the inflammasome mediating the release of IL-1β in a migraine-model [30]. We speculate that fatigue and headache are somewhat regulated by the NLRP3 inflammatory process. We hypothesize that our finding of a clustering of some AEs with headache is possibly explained by such an activation of the innate immune system, which is the first line of defense against pathogenic agents.

Research also proposed that injection site pain, type of vaccine or inflammation caused by vaccination may trigger migraine aura in vulnerable patients [31], possible mechanisms could be a lower threshold for the activation of the trigemino-vascular system in subjects with primary headaches or a stronger activation of inflammatory processes in migraine [32].

However, the International Classification of Headache Disorders, third version (ICHD-3) has not yet classified or provided diagnostic criteria to differentiate vaccine-related headaches from other types of headaches. These have been discussed and retrospective reviewed recently by us in a case series of 32 outpatients with headache that changed or recurred after Covid-19 vaccination, which suggest an involvement of the activation of the NLRP3 inflammation [33].

### Strengths and limitations

Our study is one of the largest studies looking into the headache-related AEs clusters post Covid-19 vaccination and provides some new insights. However, the study also has some limitations. Firstly, our target population consists of HCWs, 71.4% of whom were female. Therefore, it may not be representative of the general population. Secondly, the analyses featured in this paper were conducted utilizing a database derived from a previously published study. Given that the original study had specific objectives pertaining to a distinct aspect of Covid-19 vaccine epidemiology, its database hinders the exploration of various pertinent inquiries and does not facilitate the verification of participants’ migraine or headache histories, nor does it permit the confirmation of the precise timing and duration of their symptoms. Thirdly, there is a recall bias since participants tend to remember the AEs more clear after the second vaccination and that may influence the result.

## Conclusions

Our study confirmed the findings of previous studies that headaches are more frequent and more severe after the second Covid-19 vaccination. Furthermore, AEs such as fatigue, flu-like symptoms, pain at the injection site, known tension-type headache, fever, dizziness/balance problems, and known migraine attacks can predict the severity of headache. We envisaged that cluster AEs including headache post Covid-19 vaccination might share a basic mechanism. These findings provide new insights for further investigation related to Covid-19 vaccinated headaches.

### Electronic supplementary material

Below is the link to the electronic supplementary material.

Supplementary Material 1

## Data Availability

The datasets used and analyzed during the current study are available on reasonable request with permission of Munich University hospital data protection office.

## Abbreviations

1st: First
2nd: Second
A: Average
AEs: Adverse Events
AUC: Area under curve
Covid-19: The Coronavirus disease 2019
HCWs: Healthcare Workers
ICHD-3: International Classification of Headache Disorders, third version
L: Little
N: Not at all
NLRP3: NOD-like receptor protein 3
Q1/Q2/Q3/Q4/Q5: Question1/ Question2/ Question3/ Question4/ Question5
Q: Quite
ROC: Receiver Operating Characteristic
Sum: Summary
V: Very
