# Supplementary material for: Questionnaire-based study of COVID-19 vaccination induced headache: evidence of clusters of adverse events
Source: BMC Neurol. 2024 Mar 2;24:84. doi: 10.1186/s12883-024-03583-6 (PMC10908065; doi:10.1186/s12883-024-03583-6)
Supplement: Supplementary file 1 — Supplementary Material 1 [file 12883_2024_3583_MOESM1_ESM.pdf]

DeepL

Subscribe to DeepL Pro to translate larger documents.
Visit www.DeepL.com/pro for more information.

LMU CLINICAL CENTER SURVEY: CORONA VACCINATION PROCESS

QUESTIONNAIRE ONLY FOR ALREADY VACCINATED EMPLOYEES

Dear Employee,

Thank you very much for participating in our survey.

A few weeks ago, the first vaccinations against COVID-19 were approved. Since then, several vaccination centers have become operational in Germany. One of these is the vaccination center at the LMU Klinikum.

Through this survey, we would like to hear from you about your perception of the immunization process at LMU Klinikum and how you would rate it. Your answers will help us to make this and future vaccination campaigns at LMU Klinikum more efficient.

Answering the questions takes about 5 min.

If you have any questions about this survey, you can contact **umfrage.katschutz@med.uni-muenchen.de**.

Your study team

There are 23 questions in this survey.

VACCINATION PROCESS IN GENERAL

To what extent do you agree with the following statements? *

Please select the applicable answer for each item:

	does not apply	rather not true	partly / partly	rather true	applies
**Even if it had taken me over 1 hour to travel to get the vaccine, I still would have taken the time to get there.**	○	○	○	○	○
**The vaccination center at the LMU Klinikum was easily accessible in terms of location.**	○	○	○	○	○
**The vaccination appointment was easy to organize.**	○	○	○	○	○
**The various stations in the vaccination center were logically arranged.**	○	○	○	○	○
**The vaccination process at LMU Hospital was generally well organized.**	○	○	○	○	○
**The registration and vaccination process were well organized.**	○	○	○	○	○

What was the wait time from the time you registered at the vaccination center until you received your vaccination? *

Please select only one of the following answers:

- ◯ Less than 10 minutes
- ◯ Between 10 and 20 minutes
- ◯ Between 20 and 30 minutes
- ◯ Over 30 minutes
- ◯ I can not remember

How long were you at the LMU Hospital Immunization Center in total? *

Please select only one of the following answers:

- ◯ Less than 30 minutes
- ◯ Between 30 and 45 minutes
- ◯ Between 45 and 60 minutes
- ◯ About 1 hour
- ◯ I can not remember

How would you rate the following aspects of the immunization process at LMU Hospital? *

Please select the applicable answer for each item:

	bad	less good	neutral	go<br>od	very good
**Prioritization of the areas to be vaccinated**	◯	◯	◯	◯	◯
**Availability of the vaccine**	◯	◯	◯	◯	◯
**Organization of the appointment booking**	◯	◯	◯	◯	◯
**Scheduling of the administration of the second vaccination dose (availability of scheduling options).**	◯	◯	◯	◯	◯
**Process of registration in the vaccination center**	◯	◯	◯	◯	◯
**Possibility of a medical consultation in the vaccination center**	◯	◯	◯	◯	◯
**Preparation of the vaccine doses**	◯	◯	◯	◯	◯
**Vaccination**	◯	◯	◯	◯	◯
**Follow-up in the vaccination center**	◯	◯	◯	◯	◯

Did you observe any side effects after the 1st vaccination? *

Please select only one of the following answers:

- ◯ Yes
- ◯ No

Which of the following vaccine reactions or side effects did you experience after the first dose of vaccine?

Please select the applicable answer for each item:

	not at all	little	medium	quite	very		Up to 72 hours after vaccination	After >72 hours after vaccination
Pain at the injection site	○	○	○	○	○		○	○
Redness	○	○	○	○	○		○	○
Diarrhea	○	○	○	○	○		○	○
Nausea, vomiting	○	○	○	○	○		○	○
Fever (≥ 38.0°C)	○	○	○	○	○		○	○
Circulation weakness (e.g. "black" before the eyes)	○	○	○	○	○		○	○

**Dizziness/ Balance problem**	◯	◯	◯	◯	◯		◯	◯
	**not at all**	**little**	**medium**	**quite**	**very**		**Up to 72 hours after vaccination**	**After >72 hours after vaccination**
**Known tension headache (triggering an attack within 24h)**	◯	◯	◯	◯	◯		◯	◯
**Known migraine (triggering of an attack within 24h)**	◯	◯	◯	◯	◯		◯	◯
**Headache**	◯	◯	◯	◯	◯		◯	◯
**Flu-like symptoms**	◯	◯	◯	◯	◯		◯	◯

**Fatigue**	◯	◯	◯	◯	◯		◯	◯
	**not at all**	**little**	**medium**	**quite**	**very**		**Up to 72 hours after vaccination**	**After >72 hours after vaccination**
**Hematoma (bruise)**	◯	◯	◯	◯	◯		◯	◯

Have you observed any other vaccination reactions or side effects up to 1 week after the first vaccination? *

Please select only one of the following answers:

◯ No

◯ Other

Did you observe any side effects after the 2nd vaccination? *

Please select only one of the following answers:

◯ Yes

◯ No

Which of the following vaccine reactions or side effects did you experience after the second dose of vaccine?

Please select the applicable answer for each item:

	not at all	little	medium	quite	very		Up to 72 hours after vaccination	After >72 hours after vaccination
**Diarrhea**	○	○	○	○	○		○	○
**Pain at the injection site**	○	○	○	○	○		○	○
**Redness**	○	○	○	○	○		○	○
**Hematoma**	○	○	○	○	○		○	○
**Fatigue**	○	○	○	○	○		○	○
**Flu-like symptoms**	○	○	○	○	○		○	○

**Headache**	◯	◯	◯	◯	◯		◯	◯
	**not at all**	**little**	**medium**	**quite**	**very**		**Up to 72 hours after vaccination**	**After >72 hours after vaccination**
**Known migraine (triggering of an attack within 24h)**	◯	◯	◯	◯	◯		◯	◯
**Known tension headache (triggering an attack within 24h)**	◯	◯	◯	◯	◯		◯	◯
**Dizziness/ Balance problems**	◯	◯	◯	◯	◯		◯	◯
**Circulations weakness (e.g. "black" before the eyes)**	◯	◯	◯	◯	◯		◯	◯

Fever (≥ 38.0°C)	○	○	○	○	○		○	○
	not at all	little	medium	quite	very		Up to 72 hours after vaccination	After >72 hours after vaccination
Nausea, vomiting	○	○	○	○	○		○	○

Have you observed any other vaccination reactions or side effects up to 1 week after the second vaccination? *

Please select only one of the following answers:

○ No

○ Other

VACCINATION PROCESS | INFORMATION

How would you rate the following sources of information as part of the immunization process? *

Please select the applicable answer for each item:

	not helpful at all	only conditionally helpful	somewhat helpful	very helpful	extremely helpful
**Information possibility via the e-mail address corona.impfung@med.uni-muenchen.de**	◯	◯	◯	◯	◯
**Information by doctors in the vaccination center**	◯	◯	◯	◯	◯
**Information from the LMU Hospital (e.g. intranet, newsletter)**	◯	◯	◯	◯	◯
**Notes for reconnaissance**	◯	◯	◯	◯	◯
**Data protection consent of the employee willing to be vaccinated**	◯	◯	◯	◯	◯
**Information leaflet on vaccination against SARS-CoV-2**	◯	◯	◯	◯	◯
**Medical history form and vaccination consent form**	◯	◯	◯	◯	◯

Did you request an educational interview prior to vaccination? *

Please select only one of the following answers:

- ◯ Yes
- ◯ No

To what extent do you agree with the following statements? *

Please select the applicable answer for each item:

	does not apply	rather not true	partly / partly	rather true	applies
**The doctor was trustworthy.**	◯	◯	◯	◯	◯
**The doctor has taken enough time for the educational discussion.**	◯	◯	◯	◯	◯
**The information provided by the doctor was understandable for me.**	◯	◯	◯	◯	◯
**The doctor was able to answer all my questions.**	◯	◯	◯	◯	◯

Do you know who to contact if you experience side effects from the vaccination or complications after the vaccination process? *

Please select only one of the following answers:

- ◯ **Yes**
- ◯ **No**

VACCINATION PROCESS | SAFETY

To what extent do you agree with the following statements? *

Please select the applicable answer for each item:

	does not apply	rather not true	partly / partly	rather true	applies
I generally felt safe during my stay at the vaccination center.	○	○	○	○	○
I felt like I was well taken care of even in an emergency during the vaccination process.	○	○	○	○	○
I found the follow-up period useful.	○	○	○	○	○
I found the follow-up time to be sufficient.	○	○	○	○	○
My privacy was well protected throughout the vaccination process.	○	○	○	○	○

HEALTH BEHAVIOR

To what extent do you agree with the following statements? *

Please select the applicable answer for each item:

	does not apply	rather not true	partly / partly	rather true	applies
**Although I am vaccinated, I will continue to follow the hygiene rules in private.**	○	○	○	○	○
**Although I am vaccinated, I will get tested for COVID-19 infection if needed.**	○	○	○	○	○
**After vaccination, I am less afraid of contracting SASR-CoV-2 in the area of my workplace than I was before vaccination.**	○	○	○	○	○
**Although I am vaccinated, I will continue to follow the hygiene rules in the professional environment.**	○	○	○	○	○
**Despite the vaccination campaign, the hygiene rules (e.g., mandatory MNS) are to apply at LMU Klinikum until the end of 2021.**	○	○	○	○	○

**Despite the vaccination campaign, testing opportunities at LMU Hospital are expected to continue.**	○	○	○	○	○

	**does not apply**	**rather not true**	**partly / partly**	**rather true**	**applies**
**After the vaccination, I am less afraid of contracting COVID-19 in private than before or less afraid for my relatives. than before the vaccination.**	○	○	○	○	○
**Despite the vaccination campaign, the hygiene rules (e.g., mandatory MNS) at LMU Hospital will continue to apply in 2022.**	○	○	○	○	○

GENERAL INFORMATION

At which location do you operate? *

Please select only one of the following answers:

- ◯ **Großhadern**
- ◯ **Downtown Both**
- ◯ **and Other**
- ◯

In which organizational unit/personnel area do you regularly work? (voluntary information)

Please select only one of the following answers:

- ◯ Dept. of Construction and Technology
- ◯ Dept. Procurement & Economics
- ◯ Dept.
- ◯ Dept. of Medical Technology
- ◯ and IT Dept. of Patient
- ◯ Management Department of
- ◯ Vascular Surgery
- ◯ Department of Hand, Plastic and Aesthetic Surgery Department
- ◯ of Infectious and Tropical Medicine
- ◯ Department of Pediatric Cardiology and Pediatric Intensive
- ◯ Care Med. Division of Clinical Pharmacology
- ◯ Department of Thoracic Surgery
- ◯ Department of Transfusion Medicine, Cell Therapeutics and
- ◯ Hem. Eye Clinic and Polyclinic
- ◯ Comprehensive Cancer Center
- ◯ Women's Hospital GH
- ◯ Gynecological Clinic
- ◯ IN Friedrich-Baur-

Institute

Cardiac Surgery Clinic and Polyclinic

- IFB - German Dizziness Center
- Inst.Schlaganf./Demenzfors.ISD
- Inst.f.Molek.Muskul.Forschung
- Inst.f.Psych.Phenomik &Genomik
- Institute of General Medicine
- Institute for Surgical Research
- Institute for Diagnostic and Interventional Neuroradiology
- Institute for Didactics and Training Research in Medicine
- Institute for Human Genetics
- Institute for Clinical Neuroimmunology
- Institute for Laboratory Medicine
- Institute for Emergency Medicine and Medical Management
- Institute for Prophylaxis and Epidemiology
- Institute and Polyclinic for Occupational, Social and Environmental Medicine
- Pediatric Surgical Clinic and Polyclinic in the Dr. von Haunerschen
- Children's Hospital and Children's Polyclinic at Dr. von Hauner's Children's Hospital
- Children's Palliative Center
- Clinic for General, Visceral and Transplant Surgery
- Clinic for General, Trauma and Reconstructive Surgery
- Clinic for Anesthesiology
- Clinic for Oral and Maxillofacial Surgery Clinic for
- Radiology

○ **Clinic and Polyclinic for Dermatology and Allergology**

○ **Department of Otorhinolaryngology** ○ **Department of Child and Adolescent Psychiatry**

○ **Clinic and Polyclinic for Nuclear Medicine**

○ **Clinic and Polyclinic for Orthopedics, Physical Medicine and Rehabilitation.** ○ **Clinic and Polyclinic for Palliative Medicine**

○ **Clinic and Polyclinic for Psychiatry and Psychotherapy**

○ **Department of Radiation Therapy and Radiation Oncology** ○ **Clinical Microbiology and Hospital Hygiene**

○ **LIFE - Center**

○ **Medical Clinic and Policlinic I** ○ **Medical Clinic and Policlinic II** ○ **Medical Clinic and Policlinic III** ○ **Medical Clinic and Policlinic IV** ○ **Medical Clinic and Policlinic V**

○ **Neurosurgical Clinic and Polyclinic** ○ **Neurological Clinic and Polyclinic**

○ **Human Resources Department** ○ **Nursing Directorate**

○ **Polyclinic for Orthodontics**

○ **Polyclinic for Dental Prosthetics**

○ **Polyclinic for Dental Preservation and Periodontology**

- ◯ **Staff positions of the Executive Board**
- ◯ **Dean of Studies/Faculty of Medicine**
- ◯ **Transplant Center**
- ◯ **Urology Clinic and Polyclinic Other**
- ◯ **Central emergency room**
- ◯

Have you worked on a designated COVID-19 unit or cared for COVID-19- patients? *

Please select only one of the following answers: No

- ◯ **Other**
- ◯ [ ]

What occupational/employment group do you belong to? *

Please select only one of the following answers:

- ◯ Pharmacy staff Doctor
- ◯ Occupational Therapists Purchasing (KMD)
- ◯ Health care and nursing staff
- ◯ Midwives/maternity nurses Hygiene
- ◯ specialist
- ◯ Physiotherapists Logistics (KMD)
- ◯ Medical technical assistants
- ◯ Medical laboratory assistant
- ◯ Medical radiology assistant Medical
- ◯ assistant
- ◯ Psychologists and psychotherapists cleaning
- ◯ (KMD)
- ◯ Food Service (KMD) Student
- ◯ Assistant Technical Service
- ◯ Administrative Service
- ◯
- ◯
- ◯
- ◯

- ◯ Business and supply service Scientific
- ◯ staff Dentist Dental assistant Other
- ◯
- ◯
- ◯

What is your highest degree? *

Please select only one of the following answers:

- ◯ None
- ◯ Secondary school leaving
- ◯ certificate Middle school
- ◯ leaving certificate High school
- ◯ leaving certificate Completed
- ◯ vocational training
- ◯ University degree (Bachelor) University degree
- ◯ (Master/Diploma) University degree (Doctorate or
- ◯ higher)

I have another school/vocational qualification

Your gender *

Please select only one of the following answers:

- ◯ male
- ◯ female
- ◯ diverse

Your age *

Please select only one of the following answers:

- ◯ < 20 years
- ◯ 20 - 29 years
- ◯ 30 - 39 years
- ◯ 40 - 49 years
- ◯ 50 - 59 years
- ◯ 60 - 69 years
- ◯ > 70 years No
- ◯ specification

Thank you for your participation!

Your study team

Submission of your completed questionnaire:
Thank you for responding to the questionnaire.
